# Breakpoints in complex chromosomal rearrangements correspond to transposase-accessible regions of DNA from mature sperm

**DOI:** 10.1007/s00439-023-02591-9

**Published:** 2023-08-24

**Authors:** Takeshi Sugimoto, Hidehito Inagaki, Tasuku Mariya, Rie Kawamura, Mariko Taniguchi-Ikeda, Seiji Mizuno, Yukako Muramatsu, Ikuya Tsuge, Hirofumi Ohashi, Nakamichi Saito, Yuiko Hasegawa, Nobuhiko Ochi, Masatoshi Yamaguchi, Jun Murotsuki, Hiroki Kurahashi

**Affiliations:** 1 Division of Molecular Genetics, Center for Medical Science, Fujita Health University, 1-98 Dengakugakubo, Kutsukake-cho, Toyoake, Aichi 470-1192 Japan; 2 Kobe Motomachi Yume Clinic, Kobe, Japan; 3 Department of Clinical Genetics, Central Hospital, Aichi Developmental Disability Center, Aichi, Japan; 4 Department of Pediatrics, Nagoya University Graduate School of Medicine, Aichi, Japan; 5 Department of Pediatrics, Fujita Health University, Aichi, Japan; 6 Division of Medical Genetics, Saitama Children’s Medical Center, Saitama, Japan; 7 Shin-Koga Hospital, Kurume, Japan; 8 Department of Medical Genetics, Osaka Women’s and Children’s Hospital, Izumi, Japan; 9 Department of Pediatrics, Aichi Prefectural Mikawa Aoitori Medical and Rehabilitation Center for Developmental Disabilities, Okazaki, Japan; 10 Department of Obstetrics and Gynecology, Faculty of Medicine, University of Miyazaki, Miyazaki, Japan; 11 Department of Maternal and Fetal Medicine, Miyagi Children’s Hospital, Sendai, Japan

## Abstract

Constitutional complex chromosomal rearrangements (CCRs) are rare cytogenetic aberrations arising in the germline via an unknown mechanism. Here we analyzed the breakpoint junctions of microscopically three-way or more complex translocations using comprehensive genomic and epigenomic analyses. All of these translocation junctions showed submicroscopic genomic complexity reminiscent of chromothripsis. The breakpoints were clustered within small genomic domains with junctions showing microhomology or microinsertions. Notably, all of the de novo cases were of paternal origin. The breakpoint distributions corresponded specifically to the ATAC-seq (assay for transposase-accessible chromatin with sequencing) read data peak of mature sperm and not to other chromatin markers or tissues. We propose that DNA breaks in CCRs may develop in an accessible region of densely packaged chromatin during post-meiotic spermiogenesis.

**Supplementary Information:**

The online version contains supplementary material available at 10.1007/s00439-023-02591-9.

## Introduction

Complex chromosomal rearrangements (CCRs) are defined as rare structural chromosomal aberrations characterized by more than two breakpoints located in one or more chromosomes (Madan 2013). This definition is based on cytogenetic microscopic examination. Cytogenetic CCRs mainly include a three-way translocation that requires three DNA breaks and involves three chromosomes, and their simple form comprises the most common group (30–45%) (Pellestor et al. 2011; Madan 2012). The developmental mechanism underlying these CCRs remains to be resolved. Recent technical advances in comprehensive molecular genetic analysis methods such as microarrays and next generation sequencing (NGS) have uncovered many of the molecular pathways leading to these complex structural rearrangements. One of the seminal discoveries in this regard was chromothripsis, which is caused by CCRs comprising numerous chromosomal breakages and random reassembly in limited regions of the chromosome. Chromothripsis was initially detected in cancer cells, but was later reported in germlines also (Stephens et al. 2011; Kloosterman et al. 2011). Mechanistic models of chromothripsis development have been proposed involving micronuclei and telomere breakages (Zhang et al. 2015; Maciejowski et al. 2015). Further to this, and based on characteristics such as the number of DNA breaks and the number of involved chromosomes, chromoanasynthesis and chromoplexy are other classes of CCRs that have also been proposed, and in combination with chromothripsis are collectively referred to as chromoanagenesis (Liu et al. 2011; Baca et al. 2013; Holland and Cleveland 2012; Zepeda-Mendoza and Morton 2019). However, the precise mechanisms leading to the formation of these CCRs have yet to be elucidated.

It has been demonstrated using NGS that the simple microscopic chromosomal rearrangements observed using conventional G-band analysis, such as translocations and inversions, actually carry submicroscopic complex structural abnormalities (Chiang et al. 2012). In this present study, we analyzed CCRs of mainly three- or four-way translocations by microscopic examination, and investigated their breakpoint junctions using comprehensive genomic and epigenomic analyses, to further elucidate the mechanisms underlying their development. Our results demonstrated that all of the de novo CCR cases were of paternal origin. Notably, the breakpoint distributions corresponded specifically to the ATAC-seq read data peak of mature sperm and not to other chromatin markers or tissues. We propose that DNA breaks in CCRs might develop in an accessible region of densely packaged chromatin during post-meiotic spermiogenesis.

## Materials and methods

### Samples

A total of 14 subjects harboring 3-way or more complex translocations had been previously identified by microscopical examination and were included in our present study series. Blood samples were obtained from these 14 cases and their parents after obtaining written informed consent. Heparinized blood samples were used for G-banding, while EDTA blood samples were used for other analyses. Genomic DNA was extracted using standard procedures. This study was approved by the local Ethical Review Committee of Fujita Health University. All procedures were performed following the ethical principles for medical research from the World Medical Association Declaration of Helsinki.

### G-band analysis for determining three-way translocations

Three-way or more complex translocations were identified by standard G-banding (Table 1) (Arsham et al. 2017).

**Table 1:** Summary table of constitutional complex chromosomal rearrangement cases

Case	Karyotype^a^	Phenotype	# BPs^b^
1	46,XX,der(9)(9pter → 9q31::18q22 → 18qter),der(10)(10pter → 10p13::?::10p11.2 → 10qter),der(18)(18pter → 18q22::9q31 → 9q33::9p34.1 → 9qter)	Recurrent pregnancy loss	6
2	46,XY,t(6;15;13)(q25.1;q26.1;q22)	Pervasive developmental disorder, not otherwise specified	16
3	46,XX or XY,der(3)(3pter → 3p12::3q25 → 3q13.2::3q12 → 3p12::20p11.2 → 20pter),der(8)(8pter → 8q13::3q1?2 → 3q1?3.2::8q22 → 8qter),der(20)(3qter → 3q25::20p11.2 → 20q12::8q?13 → 8q?22::20q12 → 20qter)	Recurrent pregnancy loss	40
4	46,XX,inv(4)(p15.3q11)t(4;21;18)(p15.3;q21;q23)	Developmental delay, leukoencephalopathy	7
5	46,XY,der(1)(1pter → 1q24::6p22 → 6pter),der(6)(9pter → 9p22::6p22 → 6q21::6q23 → 6qter),der(9)(1qter → 1q24::6?q23 → 6?q21::9p22 → 9qter)	Tactile hyperacusis	12
6	46,XX,der(2)(2pter → 2q13::?),der(10)(18qter → 18q11.2::?::10p13 → 10qter),der(18)(18pter → 18q11.2::2q21.3 → 2qter)	Primary amenorrhea	17
7	46,XY,t(3;14;4)(p26;q22;q23)	Recurrent implantation failure	4
8	46,XY,der(3)(3qter → 3q26.2::12q14 → 12q14::6p22.2 → 6pter),der(6)(3qter → 3q26.2::6p22.2 → 6qter),der(12)(qter → q14::q14 → qter)	West syndrome,	19
9	46,XY,del(1)(q25.1q32.1),der(7)t(7;13)(q31.2q12.3)ins(13;1)(q22;q25.1q32.1)del(13)(q22q34),der(9)ins(9;13)(q31;q34q22),der(13)t(7;13)(q31.2;q12.3)	Cryptorchidism, Inguinal hernia, Fistula auris congenita, Squint	26
10	46,XX,ins(10;8)(p13;q23q24),ins(12;8)(p12;q21q24)	Normal	10
11	46,XY,der(5)(5pter→5p15.3::5p14→5qter),der(7)(7pter→7q22::5p14→5p15.3::11q24.2 → 11qter),der(11)(11pter → 11q24.2::7q22 → 7qter)	Myoclonic epilepsy and developmental delay	5
12	46,XY,ins(11:2)(q21:q21.1q36),del(5)(q33.1q33.3)	Developmental delay	11
13	46,XY,der(1)(18pter → 18p11.2::1p32.1 → 1qter),del(9)(p21p22), der(18)(1pter → 1p32.1::18p11.2 → 18q21.2::18q21.3 → 18qter)	Developmental delay	8
14	46,XY,der(3)ins(11;3)(p13;q27q13.2),der(10)t(10;11)(q26;p15),der(11)ins(11;3)t(10;1	Recurrent pregnancy loss	12

^a^Karyotype by G-banding

^b^Number of breakpoints by NGS

### Breakpoint analysis by next generation sequencing (NGS)

Mate-pair sequencing (MPS) or whole-genome sequencing (WGS) was used to detect the breakpoint junctions of chromosomal rearrangements such as translocations, inversions, deletions, and duplications. For sample preparation, a Nextera Mate Pair Library Preparation Kit or TruSeq DNA PCR-Free Library Preparation Kit (Illumina, San Diego, CA) was employed in accordance with the manufacturer’s protocol. All samples were sequenced using 2 × 100 bp paired-end sequencing on an NGS platform. After mapping the reads to the reference genome, discordantly mapped reads were extracted using BreakDancer or LUMPY software. All putative breakpoint junctions were confirmed by visual inspection using Integrative Genomics Viewer (IGV). Breakpoint-junction-specific PCR was also used to confirm and determine the breakpoint junctions at a nucleotide resolution. PCR was performed with appropriate primer sets using KOD One PCR Master Mix (Toyobo, Japan). PCR products were sequenced with the Sanger method on an ABI3130xl sequencer (Life Technologies, Foster City, CA). The sequence data were aligned to the human reference genome (GRCh37/hg19) using BLAT. A circos plot was generated to connect the breakpoint junctions forming the three-way translocations or CCRs (Cheong et al. 2015).

### Copy number analysis

To determine whether any large copy number alternations had occurred, we conducted Cytoscan SNP microarray analysis (Affymetrix, Santa Clara, CA). Sample preparation was performed in accordance with the manufacturers’ instructions. Regions showing copy number changes of larger than 50 kb were extracted and these copy number polymorphisms were verified using the DGV database.

### Determination of the parental origin of a chromosomal rearrangement by SNP genotyping or STR analysis

To determine if a chromosomal rearrangement was of parental origin, we used genotype information in the vicinity of the breakpoint junction. Derivative chromosome- and normal chromosome-specific long-range PCR were separately conducted using KOD One PCR Master Mix (Toyobo, Japan). All PCR products were constructed using a Nextera XT library preparation kit (Illumina) in accordance with the manufacturer's instructions. Sequencing was performed using an MiSeq sequencer via 2 × 150 bp paired‐end sequencing (Illumina). MiSeq Reporter and VariantStudio were used for determining the genotypes of the PCR products (Illumina). After confirming any deleted regions by SNP microarray analysis, we designed FAM labeled primer sets for short tandem repeat (STR) analysis in these deleted regions. Each STR locus was amplified by PCR using KOD FX Neo polymerase (Toyobo, Japan). Fragment analysis was carried out by Sanger sequencing on an ABI3130xl sequencer (ThermoFisher Scientific, Foster City, CA). The genotypes of each STR were determined using GeneMapper software (ThermoFisher Scientific).

### Sperm chromatin state at the breakpoint junctions

We examined correlations between the breakpoint distribution and the regions where peaks were detected by ChIP-seq, ATAC-seq (assay for transposase-accessible chromatin with sequencing), and MNase-seq for mature sperm, testicles, other stages of embryogenesis, oocytes, or other somatic tissues. All sequence data for this experiment were obtained from a published dataset (Supplementary Table 1). When the experiments had been performed in duplicate, the datasets were merged using bedtools v2.29.2 and were used for subsequent calculations.

We divided the human whole genome into windows of certain sizes (1, 5, and 10 kb) and plotted sequence reads obtained from ChIP-seq, ATAC-seq, and MNase-seq data. The rate of each window was then covered by the peaks from the ChIP-seq, ATAC-seq, or MNase-seq results, and the presence or absence of the DNA breakpoints in each window was sorted in each instance. We conducted univariate analysis using the Poisson regression model using the percentages of enriched regions from the ChIP-seq, ATAC-seq, or MNase-seq results as the dependent variables and the presence of DNA breaks as the objective variable in each window to calculate the risk ratio and 95% confidence intervals (95% CIs). Statistical analysis was performed using R software and the glm function, and *P* < 0.05 was determined to indicate statistical significance using the Wald test.

## Results

**Fig. 1:**
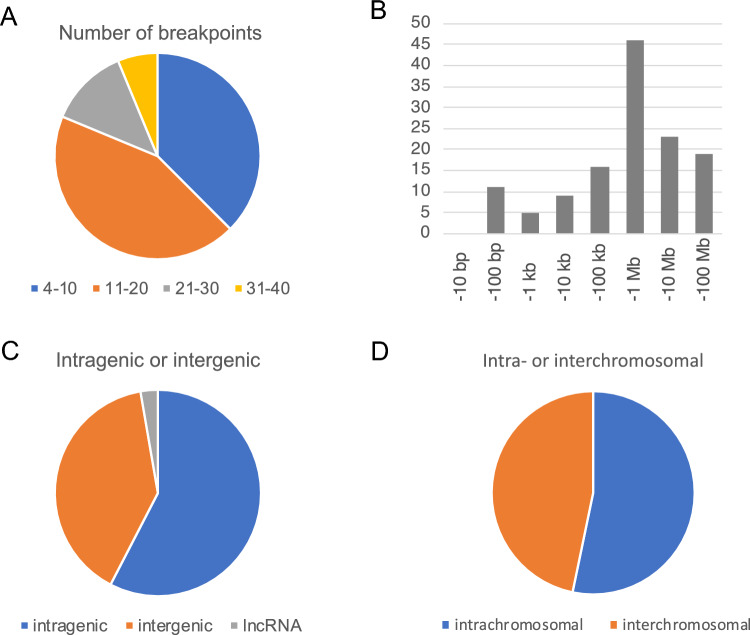
Characterization of three-way translocation breakpoints. **A** Number of breakpoints in each patient. **B** Histogram of breakpoint intervals. Fragment sizes were measured between DNA breaks. The vertical axis shows the number of interstitial fragments and the horizontal axis indicates their length. **C** Locations of the breakpoints and whether they were intragenic or intergenic. **D** Intra-chromosomal or inter-chromosomal rejoining of the DNA breaks

**Fig. 2:**
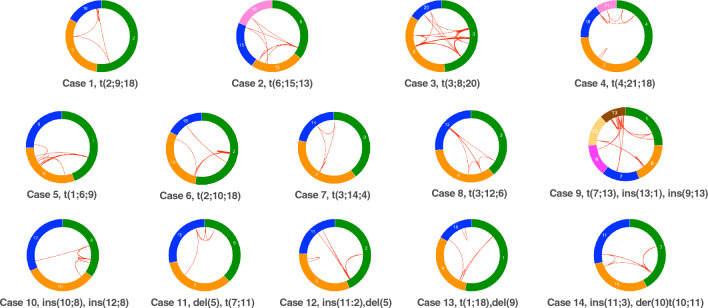
Circos visualization of genomic rearrangements. A circos plot with arcs is shown and depicts the breakpoint connections among derivative chromosomes. The colors used to denote derivative chromosome correspond to those used in the other figures

**Fig. 3:**
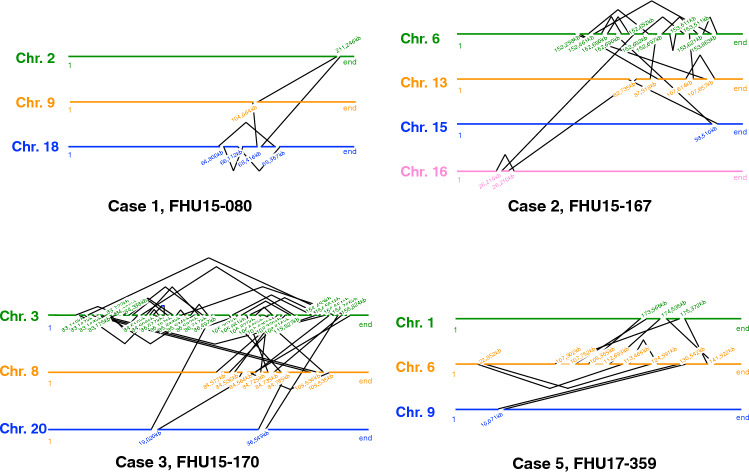
Putative structures of three-way translocations and CCRs. Illustration showing putative derivative chromosomal structures on the basis of the breakpoint junctions revealed by WGS of MPS results. Connected lines indicate breakpoint junctions. Genome positions of the breakpoint junctions were determined with reference to the human genome version GRCh37/hg19. Translucent lines indicate a deleted region

To characterize the structure of derivative chromosomes produced by three-way or more complex translocations, we utilized WGS or mate-pair sequencing (MPS) for breakpoint junction analysis, and SNP microarray for copy number analysis. We extracted discordant reads from the WGS or MPS results and confirmed them by PCR and Sanger sequencing. We did not find any simple CCRs, which indicated an exchange of more than three segments in turn among the derivative chromosomes. A total of 193 breakpoints were identified among 14 cases with CCR. The number of DNA breaks was greater than predicted by the number of derivative chromosomes, ranging from 4 to 40 in each case (Table 1, Fig. 1A). The derivative chromosomes and breakpoints involved in the chromosomal rearrangements were illustrated using a circos plot (Fig. 2). We also generated a schematic subway plot showing breakpoints of the derivative chromosomes and their genome position (Fig. 3, Supplementary Fig. 1). All of the cases had one or more cluster breakpoint regions showing a close distance between each DNA break. The median size of the intervals between two DNA breaks was 370 kb and the quartile was 2 Mb (Fig. 1B). Cases 2, 3, 5, 8, and 13 showed a multiple cluster breaks region (Fig. 2). With regard to the transcribed genes, more than half of the breakpoints were located within protein coding regions (212 in 368 breakpoints, 57.3%) (Fig. 1C).

We next analyzed how the DNA breaks were reassembled to each other. Most of the shattered fragments were recovered and reassembled with either an inverted or non-inverted orientation almost without missing any segments (Fig. 3, Supplementary Fig. 1). Of note, case 3, which involved as many as 40 breakpoints, did not show any deleted region. Only a small number of copy number losses of larger than 50 kb were identified (case 5, 12, and 13) (Supplementary Fig. 2). Three cases (case 4, 11, and 12) carried simple interstitial deletions on the chromosome not related to the translocation detected microscopically. In contrast, we did not observe any copy number gain. Since the CCRs in our present series involved more than two breakpoints in one or more chromosomes, some breakpoints were fused to others on the same chromosome (intra-chromosomal rearrangement), and on a different chromosome (inter-chromosomal rearrangement) (Fig. 1D). Notably, most of the breakpoint clusters included both intra-chromosomal and inter-chromosomal rearrangements, whereas a subset of breakpoint clusters predominantly included inter-chromosomal rearrangements. For instance, most of the breaks within the breakpoint clusters were found to be fused via an inter-chromosomal rearrangement or to a far distant region on the same chromosome in case 5 (Fig. 2).

**Fig. 4:**
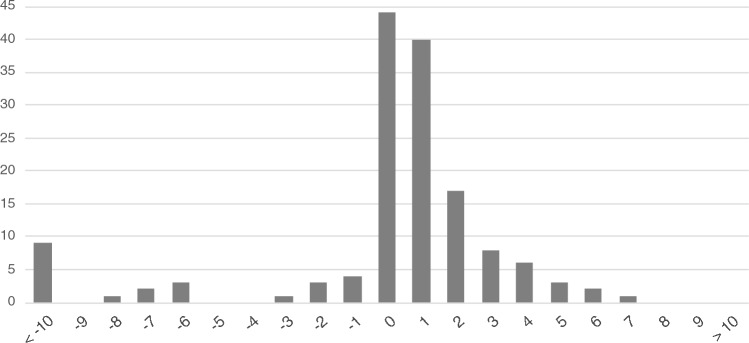
Sequence microhomology at the breakpoint junctions. The vertical axis denotes the number of breakpoint junctions. The horizontal axis indicates the length of the microhomology. Minus number indicates microinsertion. The presence of both microhomology and microinsertion suggests an MMEJ pathway as a mechanism of DNA repair

A total of 193 breakpoint junctions were validated by PCR and Sanger sequencing at a nucleotide resolution. Most of these junctions were rejoined by blunt ending or via microinsertion or microhomology, irrespective of whether the fusion was intra- or inter-chromosomal (Fig. 4). In some breakpoint junctions, small fragments of unknown origin had been inserted. Of note, the insertion observed in BP13 of case 2, BP37 of case 3, and BP11 of case 8 constituted tandem or inverted repeats with the adjacent sequence of the breakpoint, which was reminiscent of backward or serial slippages that can occur in replication-based mechanisms (Supplementary Fig. 3).

**Fig. 5:**
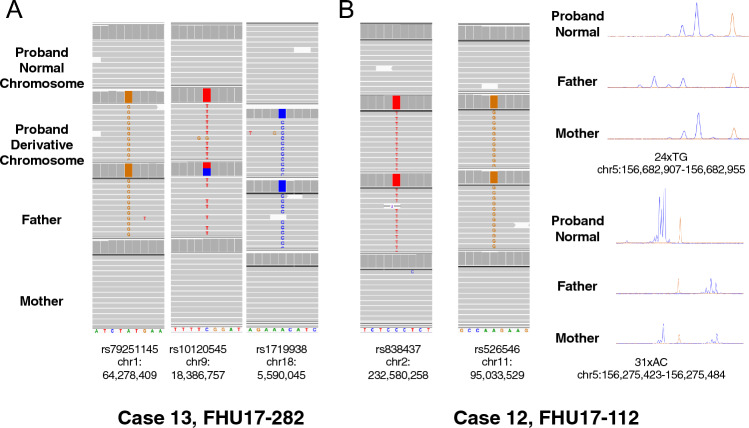
Parental origin of de novo three-way translocations. The genotypes of the proband, father, and mother are shown from top to bottom. The data indicate that all of the derivative chromosomes in de novo cases were of paternal origin. **A** Case 13. **B** Case 12

Four cases in our present study series were found to be of de novo origin, while in the remainder cases, we could not obtain parental samples. We determined a parental origin of these four de novo cases using the genotypes for the derivative and normal chromosomes near to the breakpoint junctions. All four cases were found to be of paternal origin since all of the single nucleotide variants near the breakpoints on all of the relevant chromosomes showed a paternal allele type (Fig. 5, Supplementary Fig. 4).

**Fig. 6:**
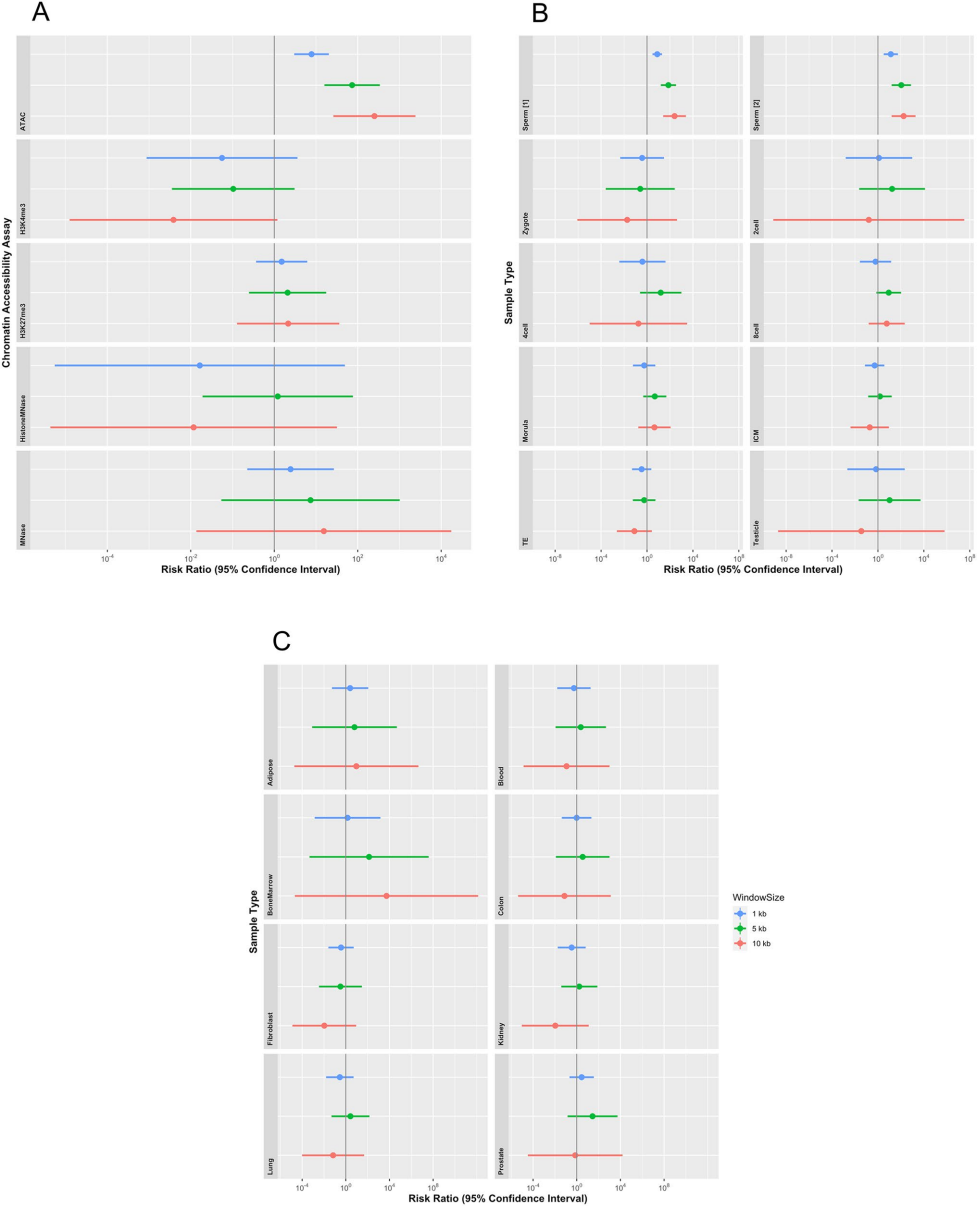
Mature sperm-specific DNA breaks in constitutional CCRs. **A** Correlations between breakpoint locations and the peaks of the sequence reads in chromatin accessibility assay for mature sperm. The data for ATAC-seq, ChIP-seq (H3K4me3, H3K27me3), Histone-MNase-seq and MNase-seq are shown. **B** Correlations between breakpoint locations and the peaks of the sequence reads in ATAC-seq for various tissues at various developmental stages of male gametogenesis and early embryogenesis. The data for mature sperm (Liu et al. 2019), zygote, four-cell stage embryo, morula, and trophectoderm are indicated in the left column, while the data for mature sperm (Jung et al. 2019), two-cell stage embryo, eight-cell stage embryo, inner cell mass, and whole testis are indicated in the right column. **C** Correlations between breakpoint locations and the peaks of the sequence reads in ATAC-seq for various somatic tissues. The data for adipose tissue, bone marrow, fibroblast, and lung are indicated in the left column, while the data for blood, colon, kidney, and prostate are indicated in the right column. Correlations were analyzed by univariate analysis using the Poisson regression model. The dependent variable and the presence of DNA breaks as the objective variable in each window were used to calculate the risk ratio and 95% confidence intervals (95% CIs). All of the database we used are shown in Supplementary Table 1 with the reference

To further analyze when and how complex rearrangements develop during male gametogenesis, we examined the correlation between the breakpoint distribution and the peak sequence reads detected by Chip-seq, ATAC-seq, and MNase-seq for tissues at various developmental stages of male gametogenesis and early embryogenesis. We first analyzed our four samples with a confirmed de novo paternal CCR origin using a Poisson regression model, but found no significant correlation between the breakpoint distribution and the peak sequence reads. However, when we used all 193 breakpoints among our 14 samples for these analyses, we observed a significantly high enrichment of the breakpoints on the chromatin accessible regions obtained from ATAC-seq of mature sperm using univariate analysis for all set window sizes (window size: 1 kb, RR, 7.8; 95% CI 3.01–20.27; *P* < 0.001; 5 kb, RR, 73.5; 95% CI 15.94–338.44; *P* < 0.001; 10 kb, RR, 251.2; 95% CI 26.10–2417.32; *P* < 0.001; Fig. 6A). In contrast, no significant enrichment was observed in other assays, indicating a chromatin status of mature sperm (ChIP-seq for H3K4me3, ChIP-seq for H3K27me3, Histone-MNase-seq and MNase-seq).

To assess whether the enrichment of breakpoints on a chromatin accessible region on ATAC-seq is specific for mature sperm, we performed similar analysis by Poisson regression for other developmental stages of male gametogenesis and early embryogenesis using samples from whole testes including spermatogonia and spermatocytes, zygotes, and early embryos. Notably, a significant correlation between the breakpoint distribution and the peak sequence reads was obtained only in mature sperm (Fig. 6B). We also examined these correlations using oocytes or other somatic tissues, but no enrichment of the breakpoints on chromatin accessible regions was observed on ATAC-seq (Fig. 6C). The oocytes could not be properly analyzed because of the small number of peak sequence reads in the data set.

## Discussion

**Fig. 7:**
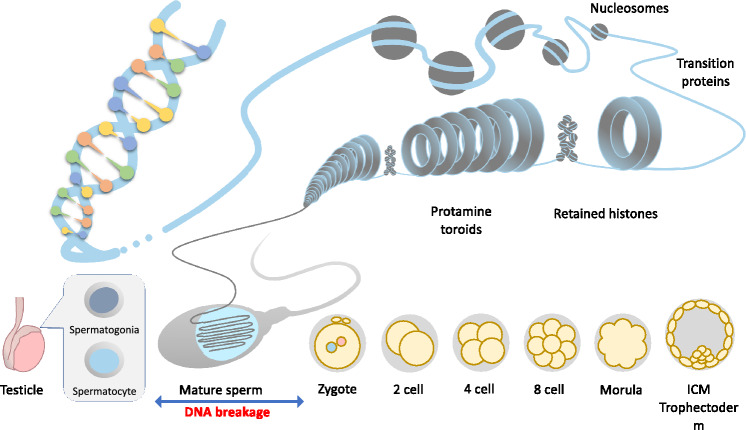
Proposed mechanism of onset of a constitutional CCR. A significantly high enrichment of the breakpoints on the accessible chromatin regions was evident from ATAC-seq data using univariate analysis, suggesting a mature sperm-specific mechanism of DNA breakage

We here conducted detailed analyses of a series of three-way or more complex translocations and found that all carried submicroscopic CCRs originating from a chromothripsis-like event. All of the de novo cases in our sample set were found to have developed in the paternal genome, which is consistent with previous studies (Pellestor et al. 2014; Fukami and Kurahashi 2018; Koltsova et al. 2019). It has been formally accepted that constitutional chromosome structural rearrangements, such as simple deletions/duplications and reciprocal translocations, are prevalently of a paternal origin, and that the age-dependent increase in their incidence suggests an involvement of DNA replication in consecutive cell divisions of pre-meiotic spermatogenesis (Hehir-Kwa et al. 2011; Brandt et al. 2019; Templado et al. 2019). Notably however, our current analyses revealed a correlation between the breakpoint location and the peak in the ATAC-seq read data obtained from mature sperm, indicating a post-meiotic origin of the CCR. The ATAC-seq methodology captures open chromatin sites and is a powerful tool for the analysis of the tissue-specific transcription regulation of each gene (Buenrostro et al. 2013; Yan et al. 2020). Mature sperm are known to have a unique chromatin configuration, i.e., the large majority (90–95%) of it is densely packaged by protamines and a smaller amount retains histones (Hammoud et al. 2009; Ward 2010). The correlation of the breakpoints with the ATAC-seq data in our present analysis suggests a mature sperm-specific DNA damage mechanism leading to the CCR (Fig. 7), although analysis of chromatin status by sequencing alone has its limitations, and it is possible that there are unknown truncations in different chromatin regions that are not detectable here.

It is generally acknowledged that DNA breaks are permissively generated and accumulated in mature sperm due to exposure to environmental mutagens, the haploid nature of the genome, an inaccessibility to DNA repair proteins, or dynamic changes in the DNA topology (Olsen et al. 2005; González-Marín et al. 2012). Our finding herein of a correlation between breakpoints and the peak of the ATAC-seq data, which indicates accessible chromatin regions, might be supported by the breakpoints distribution of mouse evolutionary genomic rearrangements to the accessible region in post-meiotic cells (Álvarez-González et al. 2022). These data may suggest that some local exposure to a small mutagen might induce clustered DNA breaks at a region not protected by histones or protamines, which are then not subsequently restored via an appropriate pathway due paradoxically to an inaccessibility of large DNA repair proteins. This contention is also supported by the larger size of the breakpoint intervals we observed in the constitutional CCRs compared to the smaller interval size seen in typical cases of chromothripsis that are often observed in cancer, and may reflect the fact that DNA in somatic cells is protected only by histones (Fig. 1B) (Malhotra et al. 2013). Three-dimensional analysis of chromosome positions in the interphase nucleus of spermatozoa shows non-random organization by chromosome size and gene density (Manvelyan et al. 2008). Integrating 3D structural analysis, such as high-resolution Hi-C, with sperm open chromatin structure may provide a better understanding of chromosome structural reconstruction.

An issue that arises from our present observation is the timing of the DNA break repair during spermatogenesis. Sperm carrying a substantial number of DNA breaks might be able to fertilize in an unrepaired state (Sakkas and Alvarez 2010). Unrepaired DNA fragments might be rejoined during the first cell cycle after fertilization when chromatin remodeling is occurring and replacing protamines with histones, i.e., toward zygotic genome activation (Gou et al. 2020). Indeed, it has been demonstrated in mice that sperm-derived DNA breaks were repaired by proteins synthesized from maternally derived mRNA after fertilization and before zygotic genome activation (Marchetti et al. 2007, 2015). DNA breaks are, thus, likely repaired during the first DNA replication that occurs within each paternal and maternal pronucleus since all of the breakpoint junctions are paternal-to-paternal. This likelihood is also supported by prior data showing that most of the DNA breaks utilize microhomology for rejoining, possibly via the microhomology-mediated end joining (MMEJ) pathway that operates in DNA repair during replication (Wood and Doublié 2016).

In conclusion, genomic and epigenomic analyses of the breakpoints of three-way or more complex translocations have provided some further clues to the mechanism underlying the onset of constitutional CCRs during post-meiotic spermiogenesis. A more thorough analysis, e.g., genomic and epigenomic analyses of DNA breaks using sperm samples derived from males accidentally exposed to mutagens, would reinforce the post-meiosis hypothesis of CCR development and might facilitate preventive approaches in the future against CCRs that can lead to congenital anomalies, neurodevelopmental diseases or recurrent reproduction failures.

### Supplementary Information

Below is the link to the electronic supplementary material.

Supplementary file1 (DOCX 26 KB)

Supplementary file2 (PDF 30 KB)

Supplementary Figure 1. Putative structures of three-way translocations and CCRs. Illustration showing putative derivative chromosomal structures on the basis of the breakpoint junctions revealed by WGS of MPS results. Connected lines indicate breakpoint junctions. Genome positions of the breakpoint junctions were determined with reference to the human genome version GRCh37/hg19. Translucent lines indicate a deleted region. Supplementary Figure 2. Copy number changes by SNP microarray analysis. Illustration showing SNP microarray analysis. Probe plots for copy numbers and B allele frequencies are shown for relevant chromosomes. Supplementary Figure 3. Sanger sequencing of the breakpoint junctions. The sequences across the flanking breakpoint junctions were determined by PCR and Sanger sequencing analysis. The microhomology sequences of the breakpoint junctions are highlighted in purple. Alignment sequences of less than 20 bp are indicated as having an unknown origin. **A**. Case 3 carries the CCR involving chromosomes 3, 8, and 20. Green characters indicate sequence of chromosome 3 origin. **B**. Case 8 carries the CCR involving chromosomes 3, 6, and 12. Blue characters indicate sequence of chromosome 12 origin. For BP37 in case 3 and BP11 in case 8, the direction of the arrow indicates the strand that is displayed. The genomic position is indicated under the arrows. Supplementary Figure 4. Parental origin of *de novo* three-way translocations. The genotypes of the proband, father, and mother are shown from top to bottom. (PDF 4779 KB)

## Data Availability

The datasets generated and analyzed in this study are available in the dbVar repository, accession number: nstd230.
