# Supplementary material for: Breakpoints in complex chromosomal rearrangements correspond to transposase-accessible regions of DNA from mature sperm
Source: Hum Genet. 2023 Aug 24;142(10):1451–60. doi: 10.1007/s00439-023-02591-9 (PMC10511381; doi:10.1007/s00439-023-02591-9)
Supplement: Supplementary file 1 — Supplementary file1 (DOCX 26 KB) [file 439_2023_2591_MOESM1_ESM.docx]

**Supplementary Table 1. List of data set acquisition sources used in the analysis.**

**Reference Supplementary Table 1.**

For germ cells

1. Liu L et al (2019) An integrated chromatin accessibility and transcriptome landscape of human pre-implantation embryos. Nat Commun 10:364.

2. Hammoud SS et al (2009) Distinctive chromatin in human sperm packages genes for embryo development. Nature 460:473-478.

3. Samans B et al (2014) Uniformity of nucleosome preservation pattern in Mammalian sperm and its connection to repetitive DNA elements. Dev Cell 30:23–35.

4. Jung YH et al (2019) Maintenance of CTCF- and Transcription Factor-Mediated Interactions from the Gametes to the Early Mouse Embryo. Mol Cell 75:154–171.e5.

5. Guo J et al (2022) Long Non-Coding RNA RFPL3S Functions as a Biomarker of Prognostic and Immunotherapeutic Prediction in Testicular Germ Cell Tumor. Front Immunol 13:859730.

For somatic cells

1. Rauch A et al (2019) Osteogenesis depends on commissioning of a network of stem cell transcription factors that act as repressors of adipogenesis. Nat Genet 51:716–727.

2. Chen X et al (2016) ATAC-see reveals the accessible genome by transposase-mediated imaging and sequencing. Nat Methods 13:1013–1020.

3. Kelso TWR et al (2016) Chromatin accessibility underlies synthetic lethality of SWI/SNF subunits in ARID1A-mutant cancers. Elife 6:e30506.

4. Piché J et al (2019) Molecular Signature of CAID Syndrome: Noncanonical Roles of SGO1 in Regulation of TGF-β Signaling and Epigenomics. Cell Mol Gastroenterol Hepatol 7:411–431.

5. Karabacak Calviello A et al (2019) Reproducible inference of transcription factor footprints in ATAC-seq and DNase-seq datasets using protocol-specific bias modeling. Genome Biol 20:42.

6. Catizone AN et al (2019) Comparison of genotoxic versus nongenotoxic stabilization of p53 provides insight into parallel stress-responsive transcriptional networks. Cell Cycle 18:809–823.

7. Raab JR et al (2019) SWI/SNF remains localized to chromatin in the presence of SCHLAP1. Nat Genet 51:26–29.
