# Supplementary material for: Breakpoints in complex chromosomal rearrangements correspond to transposase-accessible regions of DNA from mature sperm
Source: Hum Genet. 2023 Aug 24;142(10):1451–60. doi: 10.1007/s00439-023-02591-9 (PMC10511381; doi:10.1007/s00439-023-02591-9)
Supplement: Supplementary file 2 — Supplementary file2 (PDF 30 KB) [file 439_2023_2591_MOESM2_ESM.pdf]

Table 1. Summary table of constitutional complex chromosomal rearrangement cases.

Case	Karyotype[a]	Phenotype	# BPs[b]
1	46,XX,der(9)(9pter→9q31::18q22→18qter),der(10)(10pter→10p13::?::10p11.2→10qter),der(18)(18pter→18q22::9q31→9q33::9p34.1→9qter)	Recurrent pregnancy loss	6
2	46,XY,t(6;15;13)(q25.1;q26.1;q22)	Pervasive developmental disorder-not otherwise specified	16
3	46,XX or XY,der(3)(3pter→3p12::3q25→3q13.2::3q12→3p12::20p11.2→20pter),der(8)(8pter→8q13::3q1?2→3q1?3.2::8q22→8qter),der(20)(3qter→3q25::20p11.2→20q12::8q?13→8q?22::20q12→20qter)	Recurrent pregnancy loss	40
4	46,XX,inv(4)(p15.3q11)t(4;21;18)(p15.3;q21;q23)	Developmental delay, Leukoencephalopathy	7
5	46,XY,der(1)(1pter→1q24::6p22→6pter),der(6)(9pter→9p22::6p22→6q21::6q23→6qter),der(9)(1qter→1q24::6?q23→6?q21::9p22→9qter)	Tactile hyperacusis	12
6	46,XX,der(2)(2pter→2q13::?),der(10)(18qter→18q11.2::?::10p13→10qter),der(18)(18pter→18q11.2::2q21.3→2qter)	Primary amenorrhea	17
7	46,XY,t(3;14;4)(p26;q22;q23)	Recurrent implantation failure	4
8	46,XY,der(3)(3qter→3q26.2::12q14→12q14::6p22.2→6pter),der(6)(3qter→3q26.2::6p22.2→6qter),der(12)(qter→q14::q14→qter)	West syndrome,	19
9	46,XY,del(1)(q25.1q32.1),der(7)t(7;13)(q31.2q12.3)ins(13;1)(q22;q25.1q32.1)del(13)(q22q34),der(9)ins(9;13)(q31;q34q22),der(13)t(7;13)(q31.2;q12.3)	Cryptorchidism, Inguinal hernia, Fistula auris congenita, Squint	26
10	46,XX,ins(10;8)(p13;q23q24),ins(12;8)(p12;q21q24)	Normal	10
11	46,XY,der(5)(5pter→5p15.3::5p14→5qter),der(7)(7pter→7q22::5p14→5p15.3::11q24.2→11qter),der(11)(11pter→11q24.2::7q22→7qter)	Myoclonic epilepsy and Developmental delay	5
12	46,XY,ins(11:2)(q21:q21.1q36),del(5)(q33.1q33.3)	Developmental delay	11
13	46,XY,der(1)(18pter→18p11.2::1p32.1→1qter),del(9)(p21p22),der(18)(1pter→1p32.1::18p11.2→18q21.2::18q21.3→18qter)	Developmental delay	8
14	46,XY,der(3)ins(11;3)(p13;q27q13.2),der(10)t(10;11)(q26;p15),der(11)ins(11;3)t(10;11)	Recurrent pregnancy loss	12

[a]Karyotype by G-banding. [b]Number of breakpoints by NGS.
