# Supplementary material for: Breakpoints in complex chromosomal rearrangements correspond to transposase-accessible regions of DNA from mature sperm
Source: Hum Genet. 2023 Aug 24;142(10):1451–60. doi: 10.1007/s00439-023-02591-9 (PMC10511381; doi:10.1007/s00439-023-02591-9)
Supplement: Supplementary file 3 — Supplementary Figure 1. Putative structures of three-way translocations and CCRs. Illustration showing putative derivative chromosomal structures on the basis of the breakpoint junctions revealed by WGS of MPS results. Connected lines indicate breakpoint junctions. Genome positions of the breakpoint junctions were determined with reference to the human genome version GRCh37/hg19. Translucent lines indicate a deleted region. Supplementary Figure 2. Copy number changes by SNP microarray analysis. Illustration showing SNP microarray analysis. Probe plots for copy numbers and B allele frequencies are shown for relevant chromosomes. Supplementary Figure 3. Sanger sequencing of the breakpoint junctions. The sequences across the flanking breakpoint junctions were determined by PCR and Sanger sequencing analysis. The microhomology sequences of the breakpoint junctions are highlighted in purple. Alignment sequences of less than 20 bp are indicated as having an unknown origin. A. Case 3 carries the CCR involving chromosomes 3, 8, and 20. Green characters indicate sequence of chromosome 3 origin. B. Case 8 carries the CCR involving chromosomes 3, 6, and 12. Blue characters indicate sequence of chromosome 12 origin. For BP37 in case 3 and BP11 in case 8, the direction of the arrow indicates the strand that is displayed. The genomic position is indicated under the arrows. Supplementary Figure 4. Parental origin of de novo three-way translocations. The genotypes of the proband, father, and mother are shown from top to bottom. (PDF 4779 KB) [file 439_2023_2591_MOESM3_ESM.pdf]

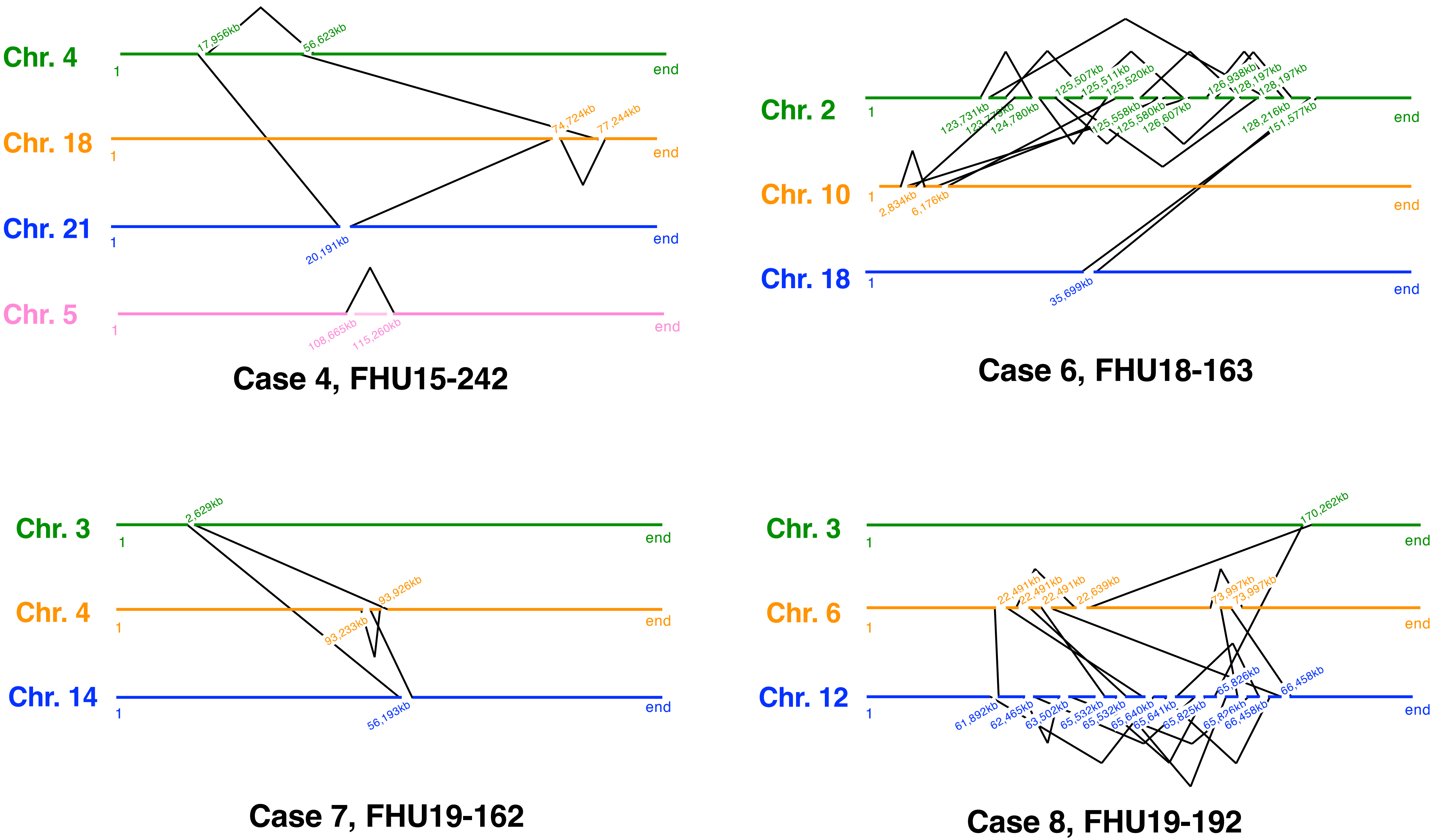

Supplementary Figure 1

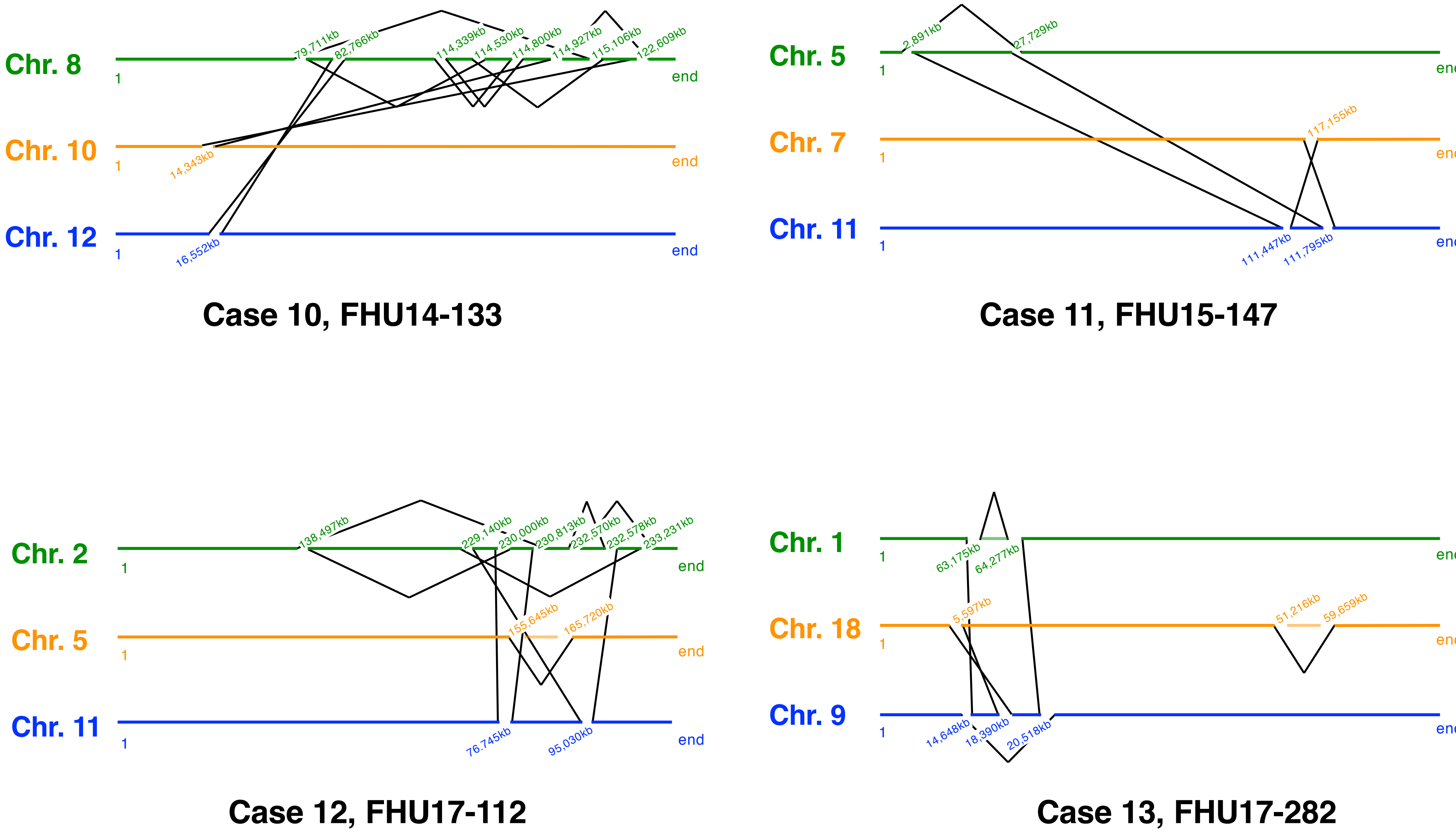

Supplementary Figure 1

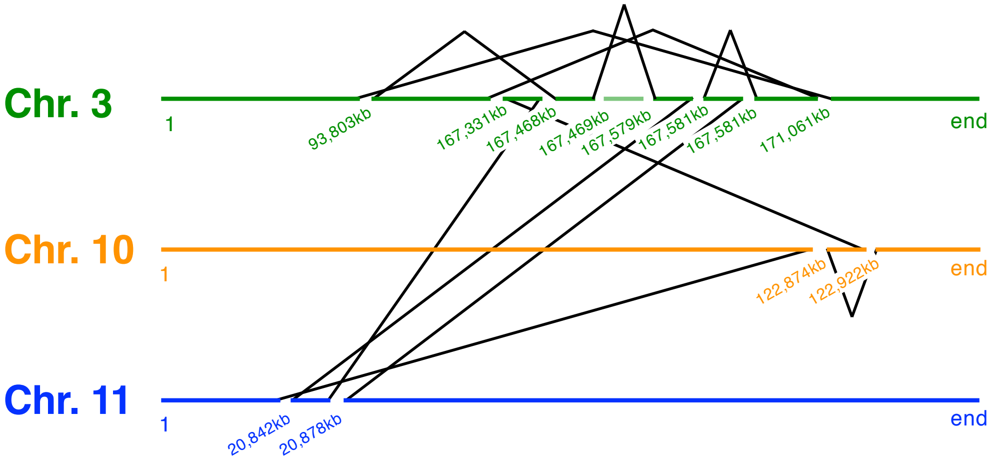

Case 14, FHU18-275

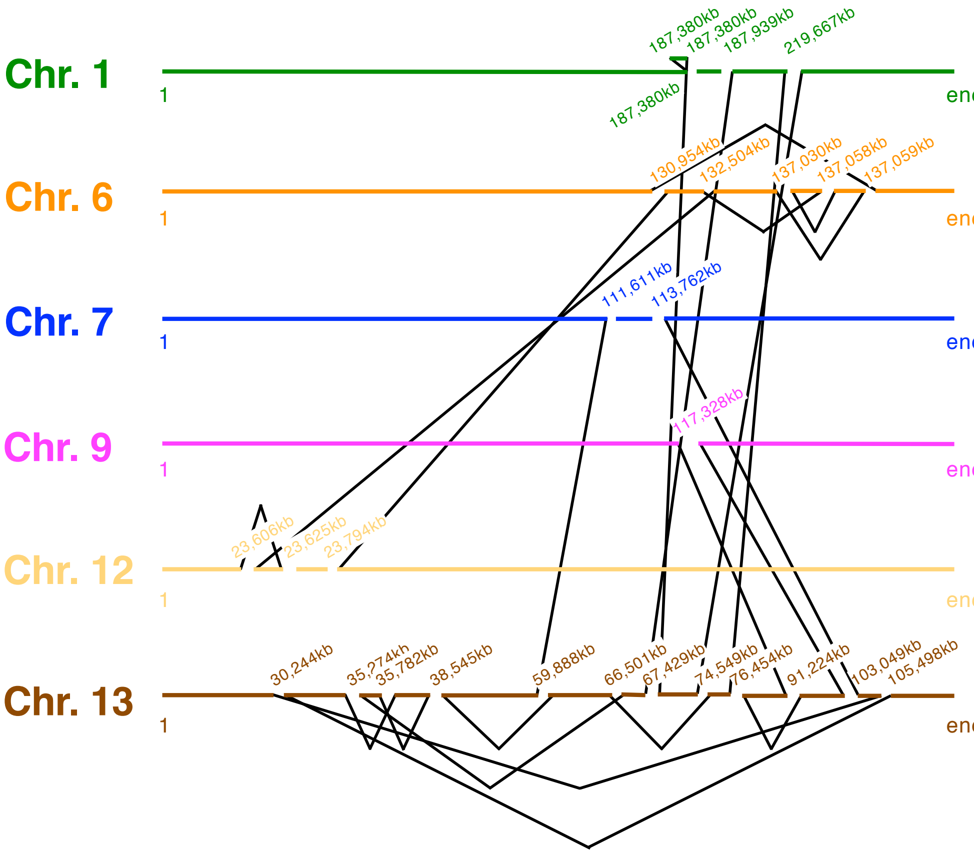

Case 9, FHU20-098

Supplementary Figure 1

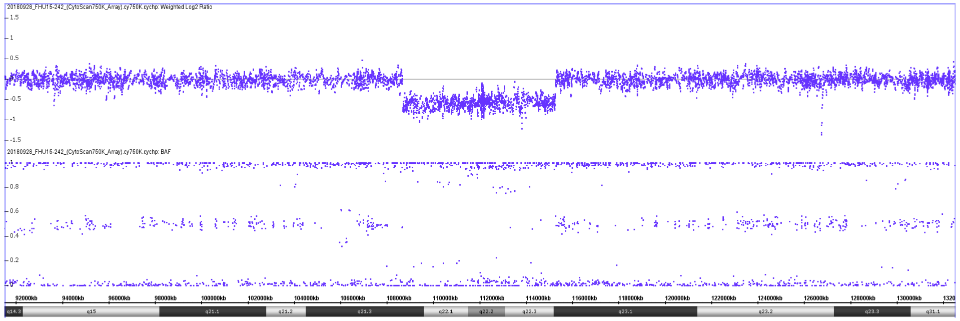

Case 4, FHU15-242, chromosome 5

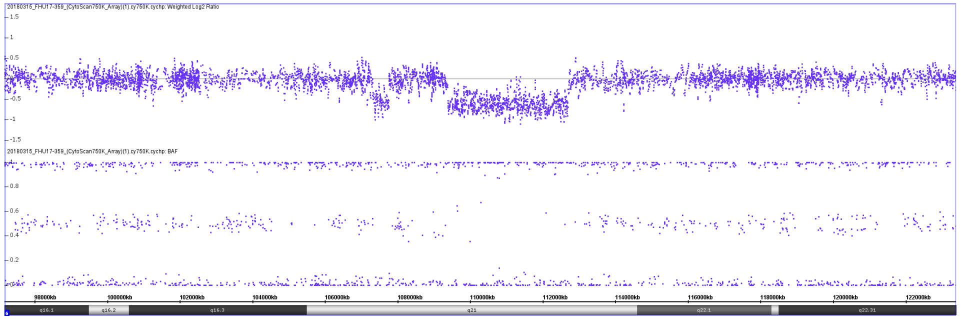

Case 5, FHU17-359, chromosome 6

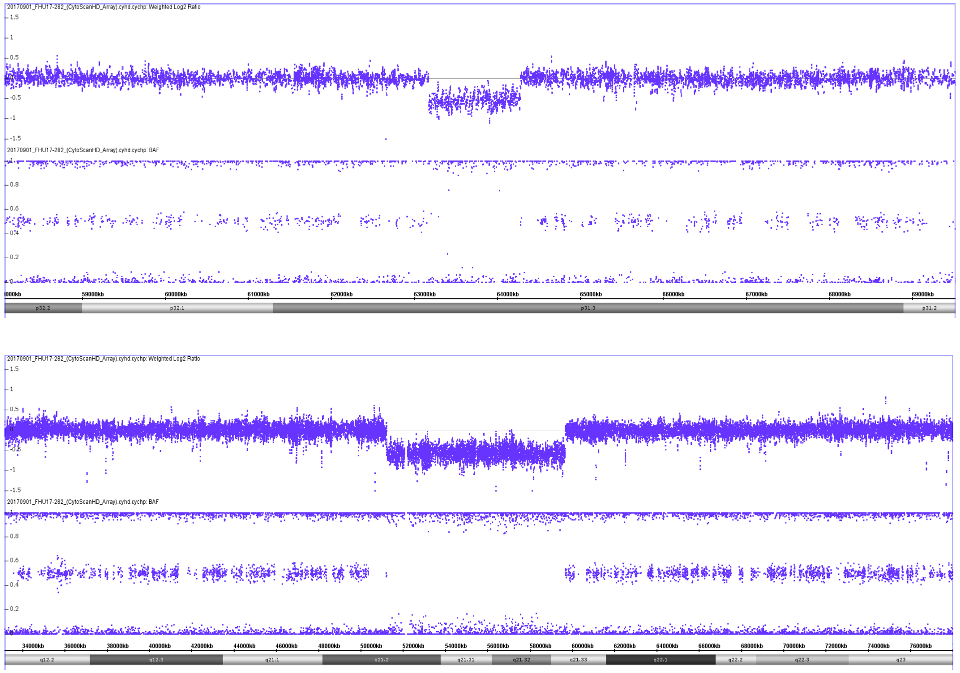

Case 13, FHU17-282, chromosome 1, 18

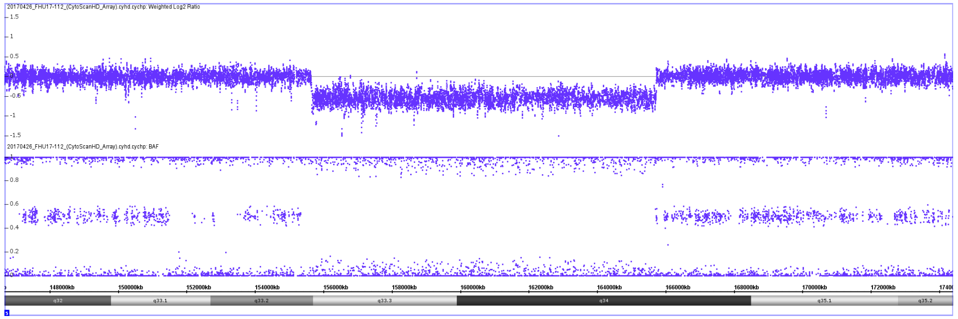

Case 12, FHU17-112, chromosome 5

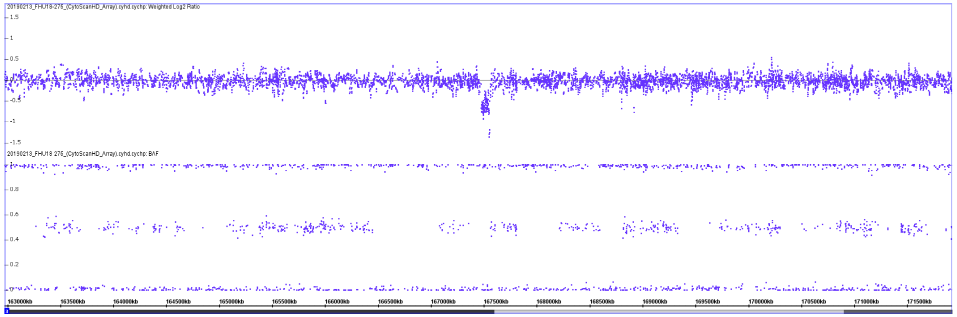

Case 14, FHU18-275, chromosome 3

Supplementary Figure 2

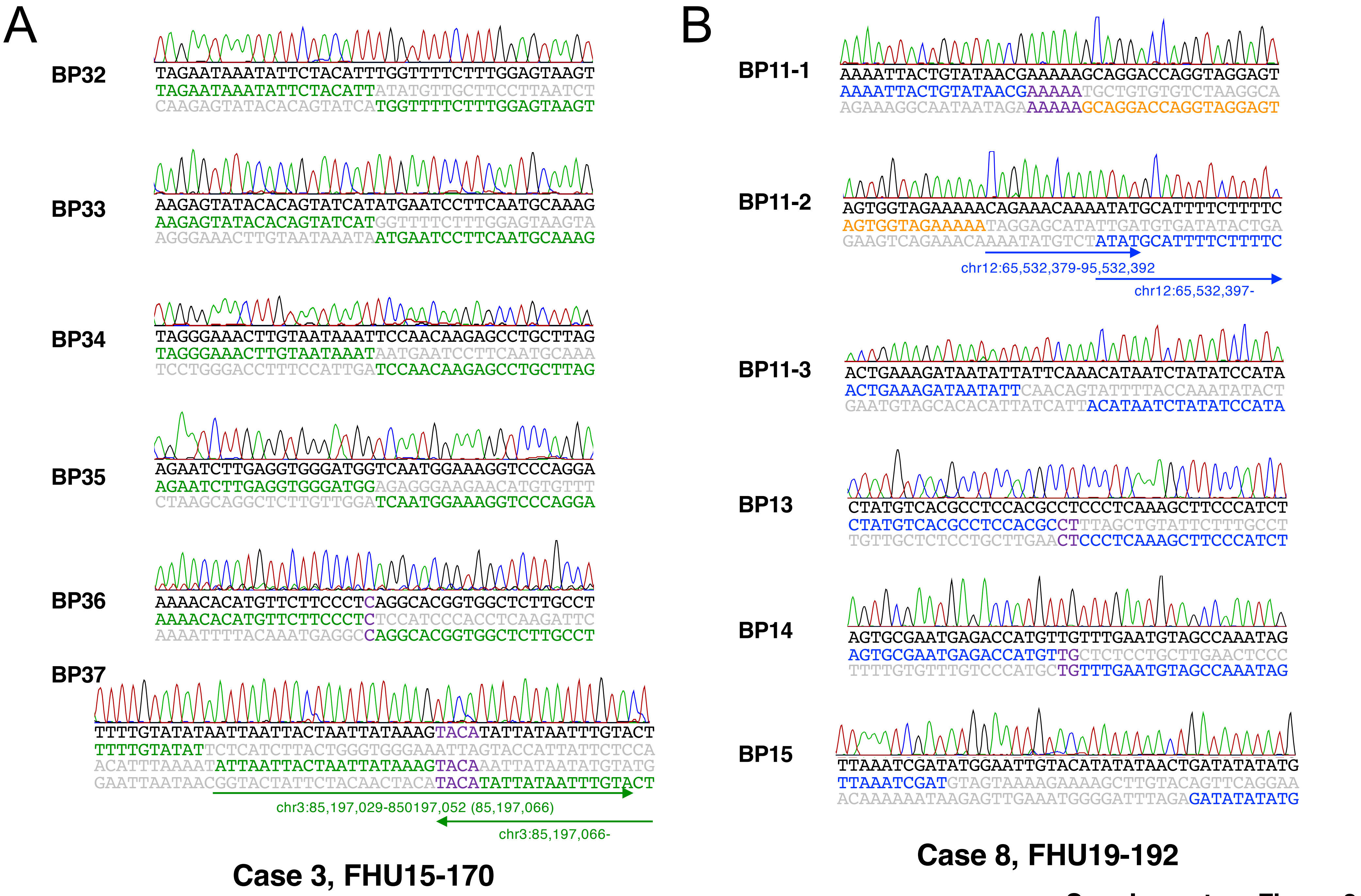

Supplementary Figure 3

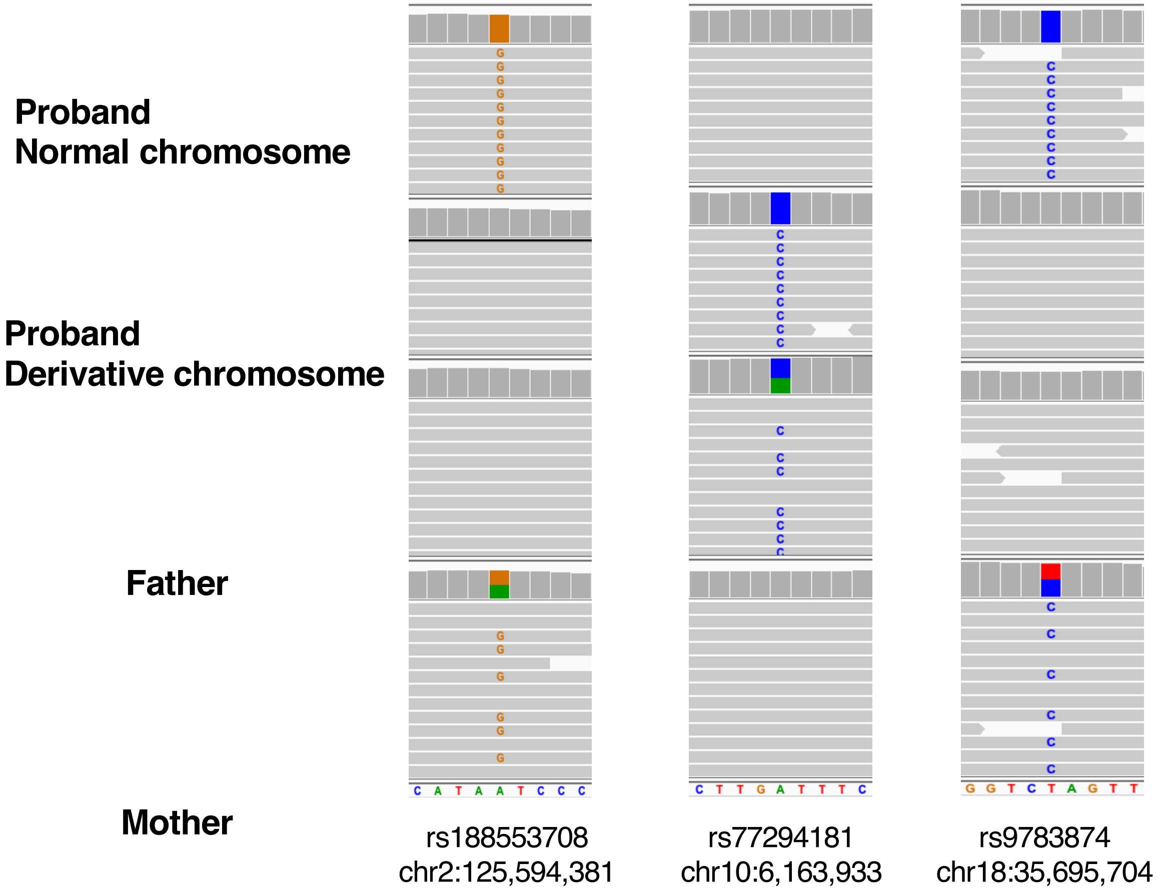

Case 6, FHU18-163

Supplementary Figure 4

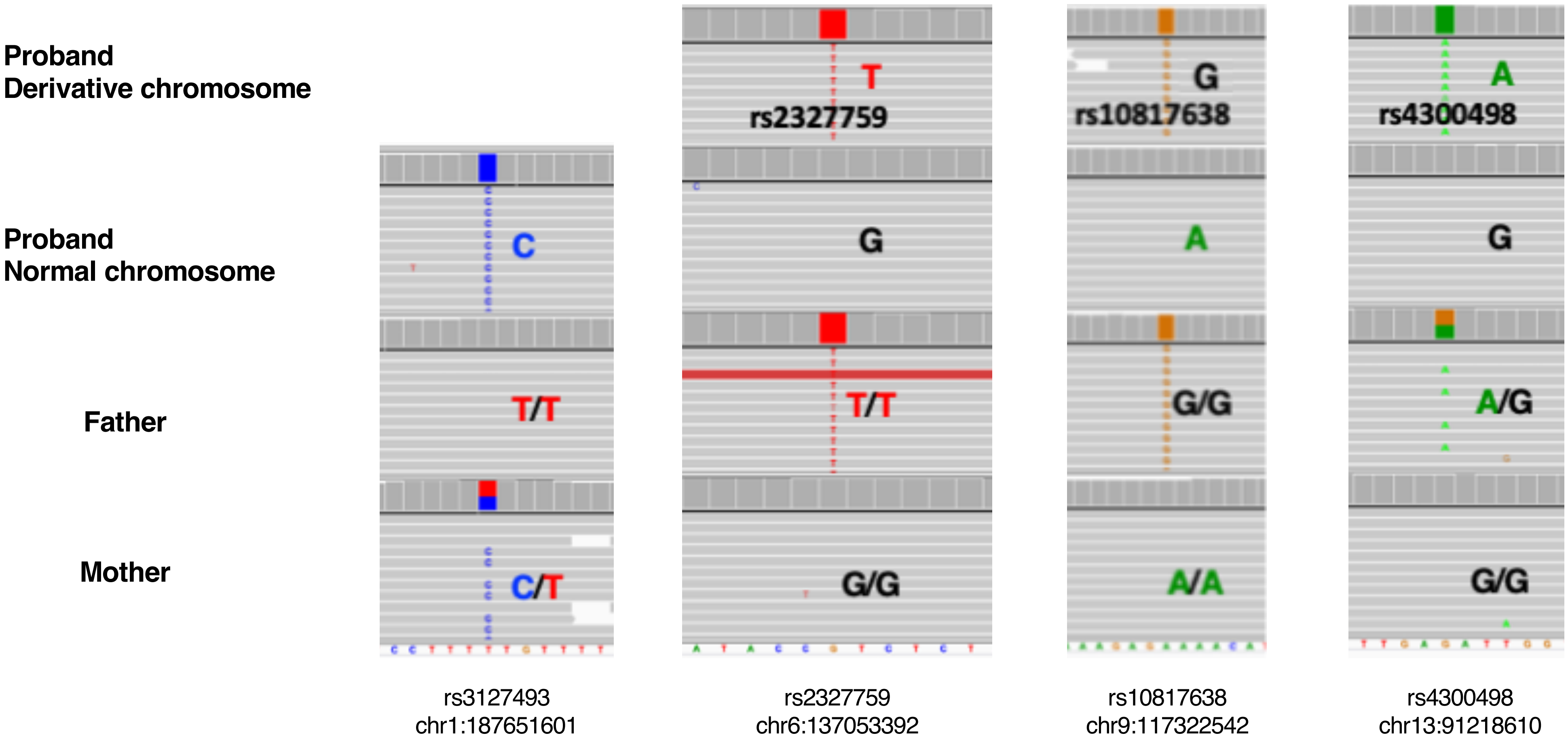

Case 9, FHU20-098

Supplementary Figure 4

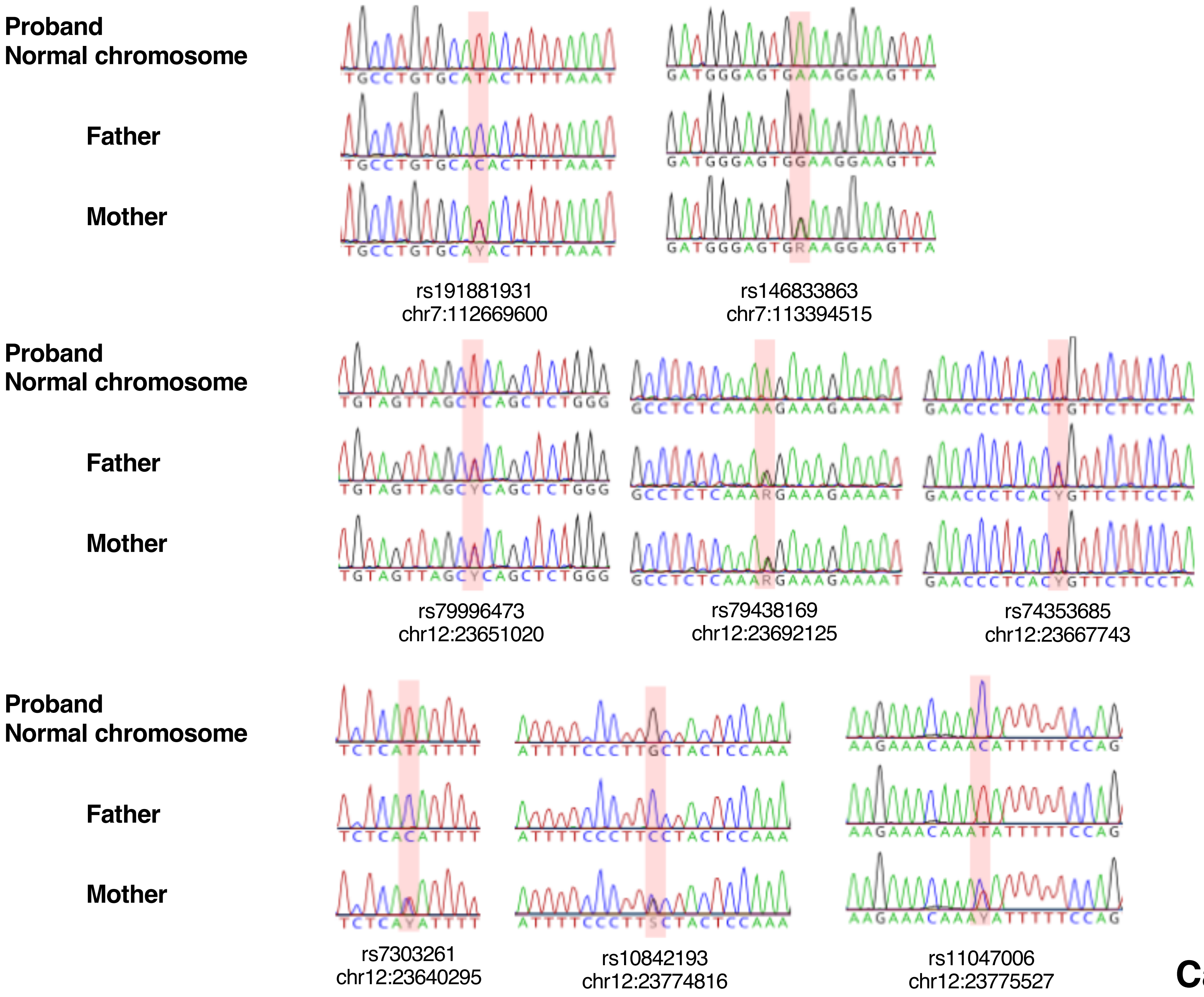

Case 9, FHU20-098

Supplementary Figure 4
